# Deciphering the Evolutionary History of Arowana Fishes (Teleostei, Osteoglossiformes, Osteoglossidae): Insight from Comparative Cytogenomics

**DOI:** 10.3390/ijms20174296

**Published:** 2019-09-02

**Authors:** Marcelo de Bello Cioffi, Petr Ráb, Tariq Ezaz, Luiz Antonio Carlos Bertollo, Sebastien Lavoué, Ezequiel Aguiar de Oliveira, Alexandr Sember, Wagner Franco Molina, Fernando Henrique Santos de Souza, Zuzana Majtánová, Thomas Liehr, Ahmed Basheer Hamid Al-Rikabi, Cassia Fernanda Yano, Patrik Viana, Eliana Feldberg, Peter Unmack, Terumi Hatanaka, Alongklod Tanomtong, Manolo Fernandez Perez

**Affiliations:** 1 Departamento de Genética e Evolução, Universidade Federal de São Carlos (UFSCar), São Carlos, SP 13565-090, Brazil; 2 Laboratory of Fish Genetics, Institute of Animal Physiology and Genetics, Czech Academy of Sciences, 27721 Liběchov, Czech Republic; 3 Institute for Applied Ecology, University of Canberra, Canberra, ACT 2617, Australia; 4 School of Biological Sciences, Universiti Sains Malaysia, Penang 11800, Malaysia; 5 Secretaria de Estado de Educação de Mato Grosso – SEDUC-MT, Cuiabá, MT 78049-909, Brazil; 6 Departamento de Biologia Celular e Genética, Centro de Biociências, Universidade Federal do Rio Grande do Norte, Natal, RN 59078-970, Brazil; 7 Institute of Human Genetics, University Hospital Jena, 07747 Jena, Germany; 8 Instituto Nacional de Pesquisas da Amazônia, Coordenação de Biodiversidade, Laboratório de Genética Animal, Petrópolis, Manaus, AM 69077-000, Brazil; 9 Toxic Substances in Livestock and Aquatic Animals Research Group, KhonKaen University, Muang, KhonKaen 40002, Thailand

**Keywords:** biogeography, DArTseq, evolution, genetic diversity, Gondwana

## Abstract

Arowanas (Osteoglossinae) are charismatic freshwater fishes with six species and two genera (*Osteoglossum* and *Scleropages*) distributed in South America, Asia, and Australia. In an attempt to provide a better assessment of the processes shaping their evolution, we employed a set of cytogenetic and genomic approaches, including i) molecular cytogenetic analyses using C- and CMA_3_/DAPI staining, repetitive DNA mapping, comparative genomic hybridization (CGH), and Zoo-FISH, along with ii) the genotypic analyses of single nucleotide polymorphisms (SNPs) generated by diversity array technology sequencing (DArTseq). We observed diploid chromosome numbers of 2*n* = 56 and 54 in *O. bicirrhosum* and *O. ferreirai*, respectively, and 2*n* = 50 in *S. formosus,* while *S. jardinii* and *S. leichardti* presented 2*n* = 48 and 44, respectively. A time-calibrated phylogenetic tree revealed that *Osteoglossum* and *Scleropages* divergence occurred approximately 50 million years ago (MYA), at the time of the final separation of Australia and South America (with Antarctica). Asian *S. formosus* and Australian *Scleropages* diverged about 35.5 MYA, substantially after the latest terrestrial connection between Australia and Southeast Asia through the Indian plate movement. Our combined data provided a comprehensive perspective of the cytogenomic diversity and evolution of arowana species on a timescale.

## 1. Introduction

The fish superorder Osteoglossomorpha is one of the three main teleostean lineages, along with Elopomorpha and Clupeocephala [1,2,3,4]. Osteoglossomorpha is divided into two orders, the relictual Hiodontiformes (comprising only two North American extant species of *Hiodon*) and the Osteoglossiformes, which currently comprises 244 valid species (restricted to freshwater tropical regions), classified into five families, namely Pantodontidae, Notopteridae, Gymnarchidae, Mormyridae, and Osteoglossidae [5,6,7]. The distribution of Osteoglossiformes is predominantly Gondwanic, with representatives occurring in Africa (center of diversity), South America, and Sahul (i.e., Australia and New Guinea) [6,7]. Such trans-oceanic distribution, combined with ancient age and distinct phylogenetic position, make Osteoglossiformes a unique model to understand the evolution and biogeography of freshwater fishes [1,6,8,9]. In this study, we focused on the subfamily Osteoglossinae of the family Osteoglossidae.

The Osteoglossidae is divided into two reciprocally monophyletic subfamilies (Osteoglossinae and Arapaiminae [= Heterotidinae]), each of them comprising two genera. The subfamily Osteoglossinae, commonly known as arowanas, includes *Osteoglossum* and *Scleropages* [5]. *Osteoglossum* is endemic to Amazonian floodplains and it currently consists of the silver arowana, *O. bicirrhosum* (Cuvier, 1829), and the black arowana, *O. ferreirai* (Kanazawa, 1966). In Southeast Asia, *Scleropages* includes two valid species [7,10], the Asian arowana *S. formosus* (Müller and Schlegel 1840) and the Batik arowana *S. inscriptus* [11]. As some allopatric populations of *S. formosus* exhibit differences in body coloration, it was thought for a long time that they might represent distinct species [12], which were formally described by Pouyaud et al. [13]. However, Kottelat and Widjanarti [14] reexamined the data from Pouyaud et al. [13] and found that they do not support the authors’ conclusions, suggesting that natural color variants were merely allopatric populations of a single species, *S. formosus* [6,7,10]. The remaining important taxonomic question is to determine whether *S. inscriptus*, which is known only from its holotype and paratype, is valid relative to *S. formosus*.

**Figure 1:**
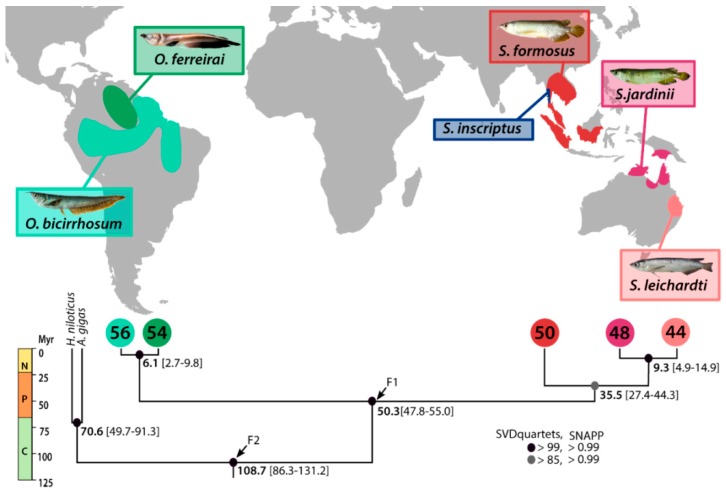
The geographic distribution of arowana, showing species color-coded by potential occurrence in South America (*O. bicirrhosum*, light green; *O. ferreirai*, dark green), Asia (*S. formosus*, red; *S. inscriptus*, blue), and Australia (*S. jardinii,* dark pink; *S. leichardti*, light pink). The consensus dated species tree given by SNAPP is shown, with estimated (average) divergence time in bold and 95% highest posterior density (HPD) intervals inside brackets for each node. Support is coded using circles in each node, and fossil calibrated nodes are indicated by arrows. Circles in the tips represent each species, with the same color used in the map, and numbers inside the circles represent the diploid chromosome number (2*n*) for each species. A geological scale with the main periods is depicted on the left (N = Neogene; P = Paleogene; C = Cretaceous).

In Sahul, *Scleropages* includes two extant species: the southern saratoga, *S. leichardti* (Günther, 1864) endemic to the Fitzroy River system in Queensland (Figure 1), and the northern saratoga, *S. jardinii* (Saville–Kent, 1892), which inhabits three separate areas in northern Australia and central-southern New Guinea. 

Previous molecular phylogenetic studies of osteoglossids were based on mitochondrial, and few nuclear markers [6,15,16,17]. These studies indicated that *Osteoglossum* and *Scleropages* were sister groups and that the same also applied for the relationship between *S. formosus* and Australian *Scleropages*. It should be noted that the monophyly of *Scleropages* has not yet been corroborated by convincing morphological evidence [18]. Whereas the molecular phylogeny of arowana seems well resolved, where several recent studies raised potential artifacts of using only one or few markers, resulting in highly supported but unreliable inferences [19,20,21]. High throughput sequencing technology (NGS) and genotyping by sequencing methods have the power to overcome the limitations of obtaining large datasets from non-model species that include genetic information from thousands of genomic regions [22,23,24]. Among the strategies available for obtaining polymorphic markers, DArTseq (diversity array technology sequencing) has increasingly been used for phylogeographic and phylogenetic purposes [25,26,27,28,29].

The extant arowana species have a discontinuous geographic distribution across South America, Southeast Asia, and Sahul (Figure 1). The causes of such disjunct distributions are still unclear, with two opposing hypotheses being debated (for a comprehensive review, see Reference [6]). Some authors assert that the biogeography of Osteoglossinae was driven by the tectonic-mediated Gondwanan fragmentation (such as the fragmentation of South America–Antarctica–Australia and/or the fragmentation of India–Australia) [8,30,31]; whereas others suggest that a post-fragmentation transmarine dispersal accounted for the observed pattern [32]. Recently published molecular dating studies tend to support a scenario in which both vicariance and marine dispersal events played a role in the distribution of these fishes [16,17]. The fossil record lends support to the marine dispersal hypothesis because it contains a fossil assigned to *Scleropages*, found in Cenozoic marine deposits [33]. Therefore, additional research is needed to test the outlined biogeographical hypotheses on the intercontinental distribution patterns of these freshwater fishes.

Modern cytogenetic and genomic tools are central to inferring the evolutionary history of ancient fish lineages (e.g., References [3,34,35,36,37,38]). Such data, however, are still scarce for Osteoglossiformes, with Osteoglossidae being a typical example, thereby limiting our understanding of their genome and karyotype evolution ([39]; reviewed in References [35,40,41,42,43]). 

Until now, the cytogenetic investigations of arowana species are limited to analyses of conventionally Giemsa-stained chromosomes, usually leading to inconsistent results. For example, Urushido et al. [44] first examined the karyotype of *S. formosus* and determined its diploid chromosome number as 2*n* = 50, while subsequent cytogenetic studies inferred 2*n* = 48 with a karyotype composed of 18 submetacentric (sm) and 30 acrocentric (a) chromosomes [40,45,46]. Karyotypes of *S. jardinii* and *S. leichardti*, were reported to have 2*n* = 48 and 2*n* = 44, respectively [47]. Suzuki et al. [48] described 2*n* = 54 in *O. ferreirai* (6 metacentric (m)-sm + 48a chromosomes), whereas its sister species, *O. bicirrhosum*, possessed a karyotype with 2*n* = 56 and 4 subtelocentric (st) + 52a chromosomes [44,48]. In a recent study by Bian et al. [40], which was partially based on molecular cytogenetic approaches, such as fluorescence in situ hybridization (FISH)-based mapping of *5S* and *18S* rDNA sites on chromosomes of *S. formosus*, the presence of a ZW sex chromosome system was supposed. 

In an attempt to provide a more complex insight into the processes shaping the evolution of arowana species, we employed a set of (molecular) cytogenetic and genomic approaches. More specifically, we applied C- and CMA_3_/DAPI staining, repetitive DNA mapping via FISH, along with interspecific comparative genomic hybridization (CGH) and Zoo-FISH (i.e., cross-species whole chromosome paintings; WCP) experiments. Time-calibrated phylogenetic reconstructions and genetic divergence were also assessed by DArTseq and were used to discuss their evolution and biogeography. Therefore, our combined dataset allowed us to provide a comprehensive perspective of the cytogenomic diversity and evolution of the arowana species on a phylogenetic and timescale context.

## 2. Results

### 2.1. DArTseq Genotyping and Genetic Relationships

**Figure 2:**
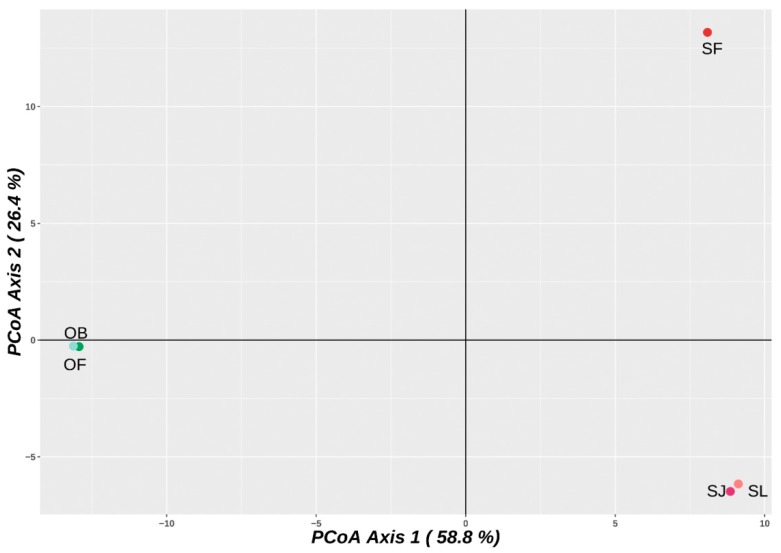
Principal coordinate analysis plot of genetic diversity in arowana species strongly corresponds to their geographical distribution. OB = *O. bicirrhosum*; OF = *O. ferreirai*; SF = *S. formosus*; SJ = *S. jardinii*; SL = *S. leichardti.*

DArTseq library preparation and sequencing resulted in 1891 high-quality filtered single nucleotide polymorphism (SNP) markers. After removing extra SNPs in reads with multiple markers, the remaining dataset contained 1565 SNPs (Supplementary Table S1). The principal coordinate analysis (PCoA) recovered 58.8% of the total variation in the first principal component (PC1), and 26.4% in the second (PC2). The analysis was able to clearly separate all arowana species located in separate continents, splitting the two analyzed genera in PC1. PC2 was able to separate the Asian and Australian *Scleropages* (Figure 2).

The species tree recovered in SVDquartets, rooted with *Arapaima gigas* and *Heterotis niloticus*, showed that *Osteoglossum* and *Scleropages* were reciprocally monophyletic. Within *Scleropages*, *S. formosus* is the sister group of Australian *Scleropages*. Bootstrap support was higher than 85% for all nodes. The script used to filter the dataset for the SNAPP analysis removed 789 monomorphic SNPs and 639 that contained missing data, resulting in a file with 463 SNPs. The species tree in SNAPP showed the same topology obtained in the SVD quartets, with all posterior probabilities equal to 1 (Figure 1). The divergence time between Arapaiminae (= Heterotidinae) and Osteoglossinae was estimated to 108.7 MYA (86.3–131.2 95% highest posterior density (HPD)). The divergence time between *Osteoglossum* and *Scleropages* was estimated to 50.3 MYA (47.8–55.0 95% HPD), in the Early Paleogene. The divergence time between *S. formosus* and Australian *Scleropages* was estimated to 35.5 MYA, in the Middle Eocene (27.4–44.3 95% HPD). The age of the crown group Australian *Scleropages* was estimated in the Neogene, 9.3 MYA (4.9–14.9 95% HPD), while the age of the crown group *Osteoglossum* was estimated to 6.1 MYA (2.7–9.8 95%HPD). All effective sample size (ESS) values were greater than 1000.

### 2.2. Karyotypes and C-Banding

**Figure 3:**
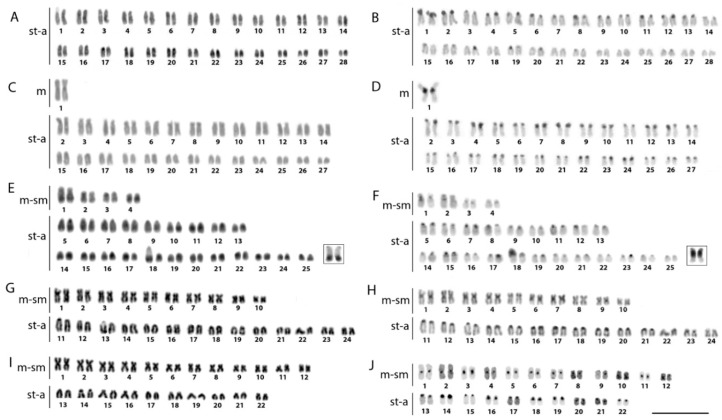
Karyotypes of the silver arowana *Osteoglossum bicirrhosum* (**A**,**B**), black arowana *Osteoglossum ferreirai* (**C**,**D**), Asian arowana *Scleropages formosus* (**E**,**F**); northern saratoga *Scleropages jardinii* (**G**,**H**) and southern saratoga *Scleropages leichardti* (**I**,**J**) arranged from Giemsa-stained (**A**,**C**,**E**,**G**,**I**) and C-banded chromosomes (**B**,**D**,**F**,**H**,**J**). In boxes (**E**,**F**), the variant forms of the 18th chromosome pair of Asian arowana in relation to its C-positive heterochromatin content (for more details, please check Section 3.2 in the discussion section) Bar = 5 µm.

Studied species differed among each other by their 2*n*. In our sampling, we did not observe any karyotype differences between males and females. Both South American arowanas displayed the highest 2*n* found among arowanas to date. While *O. bicirrhosum* exhibited 2*n* = 56, with karyotype being composed only of st-a chromosomes, *O. ferreirai* possessed 2*n* = 54, with almost all chromosomes being acrocentric, except for a large metacentric chromosome pair indicative of previous fusion events. *S. formosus* had 2*n* = 50 (8sm/m + 42st/a), and a remarkable size polymorphism was observed in the 18th pair (Figure 3E, F boxed) due to the presence of large rDNA sites (see below). The two Australian arowanas *S. jardinii* and *S. leichardti* had 2*n* = 48 and 44, respectively, and karyotypes were dominated by a high proportion of m-sm chromosomes (Figure 3J, H). Constitutive heterochromatin visualized as C-bands were present in the centromeric regions of chromosomes in the karyotypes of all species. Moreover, conspicuous C-positive blocks were observed in pair number 1 of *O. ferreirai* and *O. bicirrhosum,* and in mentioned pair number 18 of *S. formosus* (Figure 3).

### 2.3. FISH Mapping and CMA_3_ Banding

**Figure 4:**
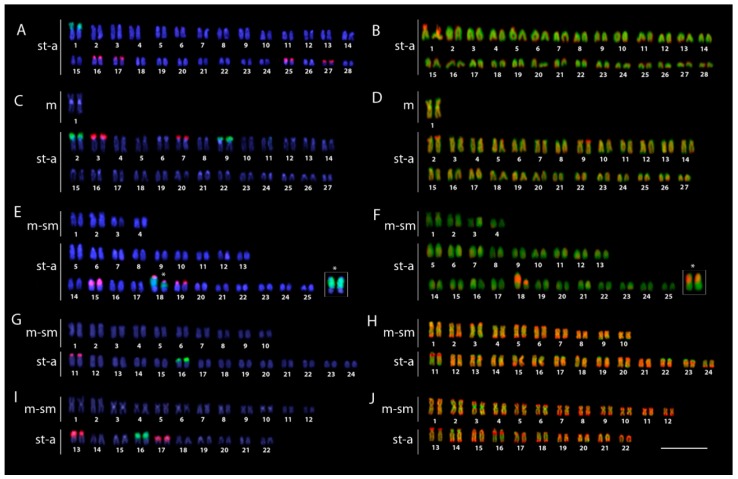
Karyotypes of the silver arowana *Osteoglossum bicirrhosum* (**A**,**B**), black arowana *Osteoglossum ferreirai* (**C**,**D**), Asian arowana *Scleropages formosus* (**E**,**F**); northern saratoga *Scleropages jardinii* (**G**,**H**) and southern saratoga *Scleropages leichardti* (**I**,**J**) arranged from chromosomes labeled with *5S* rDNA (red) and *18S* rDNA (green) probes after a dual-color FISH (**A**,**C**,**E**,**G**,**I**) and after CMA_3_/DAPI staining (**B**,**D**,**F**,**H**,**J**). In boxes, the different forms of the 18th chromosome pairs of Asian arowana with their heteromorphic *18S* rDNA sites and CMA_3_^+^ signals. Bar = 5 µm.

The *18S* rDNA sites were located in the centromeric region of a single acrocentric pair in all species examined, except for *O. ferreirai*, which contained four *18S* rDNA sites. A remarkable size polymorphism was observed in the 18th pair of male and females of *S. formosus* due to the copy number variation of DNA cistrons (Figure 4E, boxed).

The *5S* rDNA sequences were located on the p arms of acrocentric chromosome pairs, where two sites (*S. jardinii*), four sites (*O. ferreirai*, *S. leichardti*); six sites (*S. formosus*), eight sites (*O. bicirrhosum*) were observed (Figure 4). In *S. formosus*, *5S* rDNA regions were found adjacent to the *18S* rDNA region on the 18th pair.

CMA_3_^+^ bands, which represent GC-rich regions, co-localized exclusively with *18S* rDNA sites in both South American arowana species. In contrast, CMA_3_/DAPI staining of chromosomes of both Australian arowana, *S. jardinii* and *S. leichardti,* produced—besides highlighting the *18S* rDNA sites—a clear CMA_3_^+^ banding pattern along the entire portion of all chromosomes, alternating with a DAPI^+^ pattern (AT-rich regions), thereby providing clear evidence of genome compartmentalization (Figure 4). FISH with the vertebrate telomeric (TTAGGG)_n_ motif applied on the arowanas with lower 2*n*, and thus, with an indicative of the occurrence of centric fusions, revealed that hybridization signals on each telomere of all chromosomes and interstitial telomeric sites (ITS) were not detected in the chromosomes of both Australian species (Supplementary Figure S1: Metaphase plates of both Australian arowanas). 

### 2.4. Comparative Genomic Hybridization

**Figure 5:**
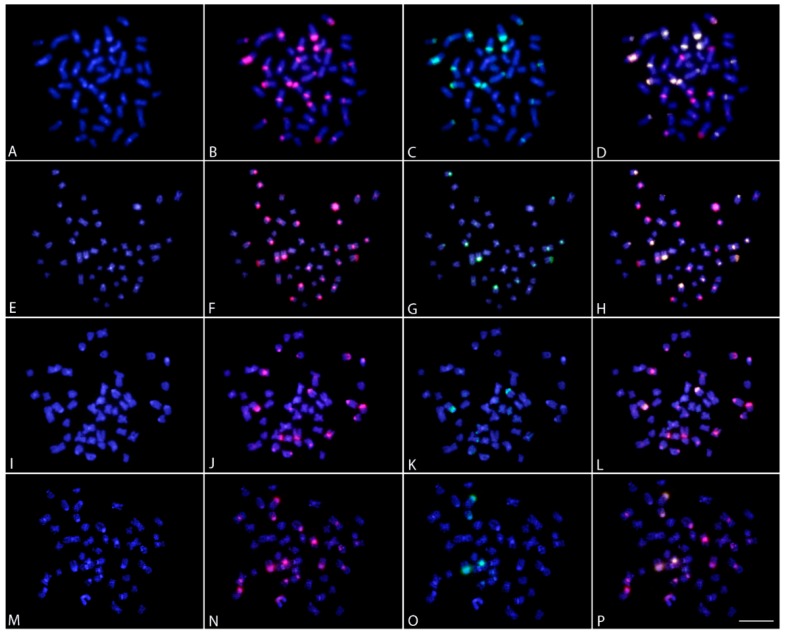
Comparative genomic hybridization (CGH) in metaphase plates of the southern saratoga *Scleropages leichardti*. First column (**A**,**E**,**I**,**M**): DAPI images (blue); Second column (**B**,**F**,**J**,**N**): hybridization pattern using *Scleropages leichardti* gDNA probe (red); Third column (**C**,**G**,**K**,**O**): hybridization pattern using the gDNA (green) of the northern saratoga *Scleropages jardinii* (**C**); Asian arowana *Scleropages formosus* (**G**); black arowana *Osteoglossum ferreirai* (**K**); silver arowana *Osteoglossum bicirrhosum* (**O**); Fourth column (**D**,**H**,**L**,**P**): merged images of both genomic probes and DAPI staining. The common genomic regions are depicted in yellow. Bar = 5 µm.

In each experiment, genome-derived probes prepared from S. *leichardti* and one of the compared species showed a rather equal binding to all chromosomes of S. *leichardti*. There was preferential localization in the centromeric and pericentromeric regions of most chromosomes and in the terminal parts of some of them (yellow signals, i.e., combination of green and red), indicating the shared repetitive content in such regions. The hybridization patterns produced by the genomic DNAs (gDNAs) of the other two *Scleropages* species (*S. jardinii* and *S. formosus*) against the *S. leichardti* chromosomal background, displayed stronger equal binding of both probes to the centromeric or telomeric regions of several chromosomes. In the cross-genera hybridization, the gDNA probes of both *Osteoglossum* species produced only a limited number of overlapping signals. More specifically, the S. *leichardti* gDNA probe hybridizing back against its own chromosome complements highlighted many heterochromatic blocks abundantly present in the centromeric and terminal chromosomal regions. Meanwhile, the probes derived from the gDNA of both *Osteoglossum* species produced only weak hybridization patterns, with few consistent signals accumulated in the terminal portions of some chromosomes and corresponding to major rDNA sites (Figure 5). The CGH experiments using gDNAs from *S. formosus* individuals carrying the conspicuous heterochromatic block in one or in both homologs of chromosomal pair number 18, demonstrated that both individuals shared the genome content and that the heterochromatic block was not accumulated with unique classes of repetitive DNA (Supplementary Figure S2: CGH on metaphase of *S. formosus*).

### 2.5. Whole Chromosome Painting of the OFE-1 Probe

**Figure 6:**
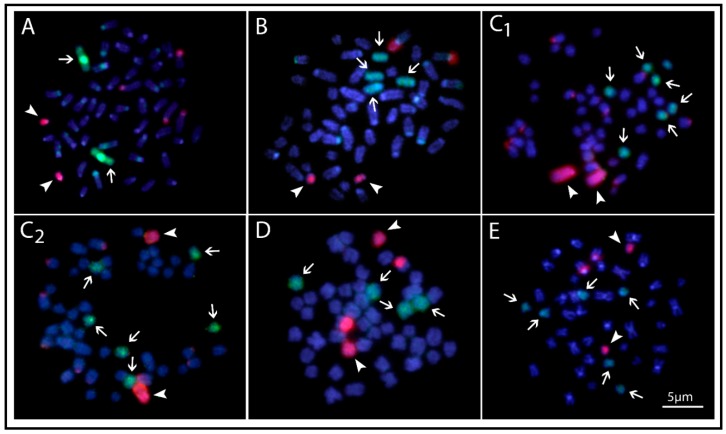
Zoo-FISH experiments with OFE-1 (arrows) and SFO-A (arrowheads) painting probes applied on the metaphase plate of the black arowana *Osteoglossum ferreirai* (**A**), silver arowana *Osteoglossum bicirrhosum* (**B**); Asian arowana *Scleropages formosus* that carries the conspicuous heterochromatic block in both (**C1**) or just one (**C2**) homologs; the Northern saratoga *Scleropages jardinii* (**D**) and Southern saratoga *Scleropages leichardti* (**E**). Bar = 5 µm.

When applied against metaphase chromosomes of *O. ferreirai,* the OFE-1 probe completely painted the first chromosome pair, with prominent hybridization signals in both centromeric regions (Figure 6A). The hybridization to other Osteoglossinae species showed that the OFE-1 probe consistently painted two (*O. bicirrhosum* and *S. jardinii*) or three (*S. formosus* and *S. leichardti*) chromosomal pairs (Figure 6). The SFO-1 probe when applied against metaphase chromosomes of *S. formosus* completely painted an acrocentric chromosome pair in individuals carrying the conspicuous heterochromatic block in both (Figure 6, C1) or just one (Figure 6, C2) of the homologs. The hybridization in the other Osteoglossidae species showed that the SFO-A probe consistently painted a small acrocentric chromosomal pair (Figure 6). Moreover, as this acrocentric pair in *S. formosus* presented a conspicuous *18S* rDNA site, additional hybridization blocks on the NOR region of some species were also observed (Figure 6).

## 3. Discussion

### 3.1. Cytogenetic Differentiation of Osteoglossum and Scleropages

Unsurprisingly, the karyotypes of *Osteoglossum* and *Scleropages* species revealed substantial macrostructural rearrangements after 50 MYA of evolutionary divergence. Higher 2*n* and karyotypes formed by acrocentric chromosomes were found in both *Osteoglossum* species compared to those of *Scleropages*, which possessed lower 2*n* and a higher number of bi-armed chromosomes. Within *Scleropages*, *S. formosus* had an intermediate pattern with 2*n* = 50 and fewer bi-armed chromosomes in the karyotype (Figure 3). While the majority of osteoglossiform species tend to maintain the karyotypes dominated by acrocentric chromosomes, both Australian arowanas and some other osteoglossiforms such as *Gymnarchus niloticus* (Gymnarchidae) and *Gnathonemus petersii* (Mormyridae) presented exceptions to this general rule ([35,39,41]; present study). The single fusion event decreasing 2*n* from 56 in *O. bicirrhosum* to 54 in *O. ferreirai* separated the karyotypes of the *Osteoglossum* species as demonstrated by Zoo-FISH. The karyotype divergence in *Scleropages* agrees with the phylogenetic hypothesis, indicating that centric fusions operated as an underlying mechanism shaping the karyotype structure, associated with reduced 2*n*, in both Australian arowanas *S. leichardti* and *S. jardinii*. Moreover, CMA_3_/DAPI staining revealed that the genomes of Australian species were demonstrably compartmentalized, similarly to those of mammals. To date, genomes compartmentalized as a mosaic of AT- and GC-rich isochores have been reported only in genera of non-teleostean gars, *Atractosteus* and *Lepisosteus,* but they were believed to be completely absent in the teleostean lineage [36]. Thus, our study brings the first evidence for such genome organization in teleosts, namely in Southeast Asian and Australian arowanas. On the other hand, South American and Southeast Asian arowanas, as a by-product of their evolutionary divergence, do not show such CMA_3_/DAPI staining patterns. *Osteoglossum* species show rDNA sites as the only GC-rich regions in the chromosomes, with the rest of the genome being stained homogeneously, indicating a balanced proportion of AT/GC composition (Figure 4). This pattern represents the most common scenario observed in extant fishes, in contrast to the one found in mammals and birds, which present a genomic GC compositional heterogeneity (reviewed in Symonová et al. [36]). Our CGH experiments compared the genomes of all arowana species, indicating that the early separation (50 MYA) produced an advanced stage of sequence divergence between *S. leichardti* and both *Osteoglossum* species, except for the NOR sites. The decrease of shared sequences as detected by CGH or related methods was expected for distantly related species and/or substantially diverged genomes [49,50,51], as also demonstrated by NGS technology using DArTseq markers (Figure 2). 

Included in the genetic divergence, the higher level of inter-chromosomal rearrangements of arowana genomes [40], herein visualized by CGH and Zoo-FISH, certainly contributed to the deterioration of karyotype/genome homogeneity between the two osteoglossid genera. Numerous structural rearrangements seem to be the predominant types of chromosomal changes among distant and close phylogenetic clades of arowanas, and they seem to be much more frequent than in some other model species (like medaka) [40]. A single chromosome fusion seemed to be the only difference between the karyotypes of both *Osteoglossum* species since the OFE-1 probe hybridized to four chromosomes in *O. bicirrhosum*. Accordingly, the karyotype found in the sister group (herein represented by *Arapaima gigas* [Arapaiminae = Heterotidinae]) was also 2*n* = 56 and formed by acrocentric chromosomes. Based on these observations, it is plausible to hypothesize that multiple chromosomal fusions took place during the karyotype evolution of the arowana species in Asia and Australia, thereby reducing the 2*n* in the other *Scleropages* species.

### 3.2. Sex Chromosomes in Scleropages formosus? 

Recently, genomic and cytogenetic analyses in the Asian arowana *S. formosus* revealed a 2*n* = 48 and the putative presence of a ZZ/ZW sex chromosome system [40]. This observation was based on the fact that the female and male karyotypes differed in the presence of one or two large chromosomes, bearing a conspicuous heterochromatic GC-rich block in the pericentromeric region, variable in length. However, the deeper cytogenetic analyses in our study clearly demonstrates that 2*n* = 50 is the correct chromosome count for this species (shared in several color variants; Cioffi, M.B. personal observation/unpublished results), corresponding to i) results of a previous study [44] and ii) the presence of 25 linkage groups in its reference genome [40,52]. On the other hand, we cannot exclude the possibility that some populations of *S. formosus* differ in their 2*n*. Moreover, our analyses focused on the putative ZW sex system in *S. formosus* but gave no support for the existence of heteromorphic sex chromosomes. Instead, the presence of size polymorphism in the 18th NOR-bearing chromosome pair was observed. A similar scenario was also observed for newts belonging to the genus *Triturus* (Amphibia, Salamandridae), where the large heteromorphic chromosome 1 was first thought to be ZW sex chromosome [53] but was later demonstrated to be related to an old balanced lethal system maintained by inversions [54]. In comparative fish cytogenetics, several studies reported the presence of sex chromosome systems based on this reasoning, but when later re-evaluated, the main cause of such observations was the presence of simple and commonly occurring size polymorphisms of the rDNA regions [55,56]. Sometimes, major rDNA sites (i.e., NOR sites) can show extensive size variation among homologs. It is widely accepted that heterochromatin polymorphisms related to such size NOR polymorphism play an important role in the sex chromosome evolution in fishes, by suppressing crossing over and triggering the initial steps of differentiation of the sex pair [57,58,59,60]. However, CGH and Zoo-Fish experiments applied in this study did not bring any evidence of accumulation of sex-specific or enriched repetitive sequences that may point to a putative sex-specific region (Figure 6, Supplementary Figure S2: CGH on metaphase of *S. formosus*).

### 3.3. Comparison of Cytogenetics of Arowana Species and Other Osteoglossiform Representatives 

The karyotype differentiation among arowana species contrasts with the evolutionary karyotype divergence found in other osteoglossiform lineages. Notopteroidei, the sister group of Osteoglossidae, whose species diverged more than 100 MYA [17], has a conserved karyotype structure and 2*n* is maintained across species over a long evolutionary time scale, with only slight disturbances of collinearity [43]. Considerable variation with regards to the number and position of rDNA sites highlights these regions as diagnostic cytotaxonomic markers for Osteoglossinae. It is well known that rDNA regions are very dynamic in fish genomes [61,62,63], evolving several times in association with rearrangements (e.g., References [64,65]).

### 3.4. Biogeography of Osteoglossinae 

The topology of our phylogenetic tree was congruent with most of the previous studies showing *Osteoglossum* as the sister group of *Scleropages*, and *S. formosus* as the sister group of Australian *Scleropages* (e.g., Reference [17]). From this topology, we could deduce that the intercontinental distribution of extant Osteoglossinae was the result of two biogeographical asynchronous events. The first event should explain the split between *Osteoglossum* (South America) and *Scleropages* (Sahul + Southeast Asia), while the second event should explain the trans-Wallace’s line distribution of *Scleropages*. There are two main hypotheses to explain the distribution of each of these intercontinental patterns (see below). One difficulty when studying the biogeography of Osteoglossinae is that the phylogeny of its extant species does not allow us to suggest the most likely region of origin of this group (see Reference [6]). The absence of any direct intercontinental connection between South America and Southeast Asia suggests that the distribution of the Osteoglossinae ancestor did not span these two regions. Consequently, the Australian region, which was directly or indirectly connected to both regions, could have played a central role in the biogeography of this group. On the other hand, there is weak evidence that the ancestral region of Osteoglossinae could include South America (but not Southeast Asia) because *Arapaima* (Arapaiminae/Heterotidinae) shares the same distribution with *Osteoglossum*. However, the fossil record does not provide clear evidence on that question because the phylogenetic positions of most of the osteoglossin fossils need to be reevaluated.

The last terrestrial connection between South America and Australia (through Antarctica) is estimated to 50–40 MYA [66]. If the divergence time between *Osteoglossum* and *Scleropages* was equal to or older than 50–40 MYA, then the vicariant hypothesis mediated by the final separation between Australia and South America cannot be rejected. Alternatively, if the divergence time is significantly younger than 40 MYA, the vicariant hypothesis will be rejected, and a marine dispersal will be preferred. Our divergence time between *Osteoglossum* and *Scleropages* (55–47.8 MYA) overlaps with the period of final contact between Australia and South America. Consequently, our results do not reject the tectonic-mediated vicariant hypothesis as the cause of the split between *Osteoglossum* and *Scleropages* [6,66].

*Scleropages* is a fully-restricted freshwater fish group found on both sides of Wallace’s line, a deep marine corridor marking the limit between the Southeast Asian and Sahul biogeographical regions. Considering the geographical distribution of *Scleropages*, we can a priori consider two hypotheses. The “Indian biotic ferry” hypothesis stipulates that the most recent common ancestor of *Scleropages* lived in Australia/India before these two continental plates separated from each other, 115–105 MYA [67]. Then, the ancestors of Southeast Asian *Scleropages* were transported by the Indian plate before dispersing to Southeast Asia after the collision between India and Eurasia. If this hypothesis is correct, the divergence time between the Southeast Asian and Australian *Scleropages* must overlap with the final fragmentation between India and Australia (approximately 105 MYA). Our results unambiguously reject this hypothesis: lineages of Southeast Asian arowana (*S. formosus*) and Australian arowanas (*S. leichardti* and *S. jardinii*) diverged from each other ~35 MYA (Figure 1), well after the separation of India and Australia. At that time, Australia was already isolated by vast marine environments, thereby making the hypothesis of marine dispersal of *Scleropages* to Southeast Asia the most likely. Our results call for a taxonomic revision of the extinct marine osteoglossomorphs and osteoglossid fossils to determine the extent to which a long-distance marine dispersal might have shaped the distribution of these fishes [9,32,68].

We note that our age estimation of *Scleropages* was substantially different from the previously proposed estimations. For example, Lavoué [16] estimated the age of the crown group *Scleropages* to a minimum of about 67 MYA (within a 95% credibility interval) and the age of the crown group Australian *Scleropages* to about 49 MYA. In our study, the divergence between *S. leichardti* and *S. jardinii* was estimated to be only 9 MYA. Heterogeneity in the evolutionary molecular rate of the mitogenomes used in Lavoué [16], as well as the distinct features of the molecular markers used herein (a high number of SNPs distributed along the genome), that are less affected by potential idiosyncrasies of using only mitogenomic regions [21], could explain most of these dating differences. Furthermore, the estimated divergence of the two outgroups used (*Arapaima* and *Heterotis*) was also more recent than estimates based on mtDNA [16,69] and was similar to recent studies with both mtDNA and nuclear markers [6,17].

The split of the South American species, *O. ferreirai* and *O. bicirrhosum*, is estimated to 6 MYA (Figure 1). The diversification of South American *Osteoglossum* species in Amazonia is coincident with the formation of the transcontinental Amazon River system over a period of about 4.9–5.6 million years through several river capture events [70]. The effects of river capture on speciation are complex, but it can subdivide the populations in new watersheds, thereby promoting the allopatric speciation [71].

The integration of cytogenetic and genomic approaches applied to the arowana species provided a clear pattern of phylogenetic relationships and evolutionary aspects of this ancient fish group. DArTseq data accessing a broad representation of the genome have revealed that the evolutionary diversification of the group is associated with both vicariant and dispersal events. The final fragmentation of southern Gondwanan regions explains the disjunct distribution of the genera *Osteoglossum* and *Scleropages*. The intercontinental distribution of species of *Scleropages* is better explained by a long-distance marine dispersal event during the late Eocene (about 35 MYA) between Australia–New Guinea and Southeast Asia.

Finally, the detailed cytogenetic survey indicated that the cytogenomic divergence patterns of Osteoglossinae were largely concordant with the inferred phylogenetic tree. Additionally, our study also enabled a precise karyotype revision leading to a correction of 2*n* and karyotype structures, as well as providing evidence of the absence of the heteromorphic ZW sex chromosome system in *S. formosus*. Further analyses are needed to detail the context of recent lineage diversification and the local adaptation of the *S. formosus* color varieties.

## 4. Materials and Methods 

### 4.1. Individuals

The number and sex of individuals investigated are presented in Table 1. The samples were collected with the authorization of the Brazilian environmental agency ICMBio/ SISBIO (License No. 48290-1) and SISGEN (A96FF09). All species were identified by morphological criteria, and voucher specimens of *S. formosus*, *S. jardinii,* and *S. leichardti* were deposited under numbers 20558, 20563, and 20564 at the Museum of Universidade Estadual Paulista (UNESP, Botucatu). The specimens of *O. bicirrhosum and O. ferreirai* were deposited at the Museum of Zoology of the University of São Paulo (MZUSP), under voucher numbers 121638 and 121640. The Australian specimens were processed as approved by the University of Canberra animal ethics committee (AEC 20180447).

### 4.2. DArTseq Genotyping and Genetic Relationships

Liver fragments of all individuals were collected and stored in 100% ethanol. The DNA extractions were performed following Sambrook and Russell [72]. Obtained DNAs were submitted to DArTseq enrichment protocol with PstI and SphI enzymes [73] and sequenced on the Illumina Hiseq2500 platform by the Diversity Arrays Technology Company (Canberra, Australia). The raw data generated by sequencing was filtered, processed, and converted to high-quality genotypes by the facility, using proprietary DArT software. Genotypes were coded as an SNP matrix with loci in the rows and individuals in the columns. For each genotype, the data was stored as 0 for reference state homozygotes, 1 for heterozygotes, and 2 for alternate state homozygotes. The R-package dartR [74] was used to filter reads with more than one SNP, leaving only the SNP with higher information content. This filtered dataset was used in all subsequent analyses. The distribution of genetic diversity between species was visualized with a PCoA, also in dartR [74].

### 4.3. Species Tree and Divergence Times

The SNP matrix was converted to the VCF format with the Radiator R-package [75] and used as input for SVDquartets [76], implemented as part of PAUP 4.0a164 [77], to estimate the species relationships with a species tree, with default parameters. The program was set to evaluate all possible quartets using 10,000 bootstrap replicates. The generated tree was visualized in FigTree 1.4.3.

The package SNAPP in BEAST 2.5.1 [78] was also used to infer the species trees and to estimate the divergence times. The XML input file was generated with the “snapp_prep.rb” script, written by Stange et al. [79]. This script prepares the input and filters the dataset, removing monomorphic SNPs and those with missing data. The analysis was carried out with two calibration points, the first one (F1 in Figure 1) in the node leading to the Osteoglossinae species (*Scleropages* and *Osteoglossum*), which was set as an exponential prior with an offset of 48.0 MYA and mean of 5.0, corresponding to the age of the earliest almost complete fossil of *Scleropages*, *S. sinensis* [80]. The second prior (F2 in Figure 1) was placed in the node marking the Osteoglossidae division time, that includes all arowana utilized as outgroups *Arapaima* and *Heterotis* (Arapaiminae = Heterotidinae). We used an exponential distribution with an offset of 72.1 MYA and mean 11.0 that was based on the oldest known crown-group osteoglossid fossil [16]. The analysis was performed using a chain length of ten million generations, with sampling at every 5000 generations. Convergence levels in the run were assessed in Tracer 1.7.1 [81]. TreeAnnotator 2.5.1 was used to infer the MCC tree based on common ancestor heights. Burn-in was set to discard the first 25% generated trees, and the consensus tree was exported in FigTree 1.4.3.

### 4.4. Chromosome Preparations, C- and CMA_3_ Banding

Mitotic chromosomes were obtained using the protocol described in Bertollo et al. [82]. The experiments followed ethical and anesthesia conducts, following the Ethics Committee on Animal Experimentation of the Universidade Federal de São Carlos (Process number CEUA 1853260315). Chromomycin A_3_ (CMA_3_, DNA dye–specific for GC-rich regions) and DAPI (AT-specific) fluorescent staining was performed as described by Schmid [83]. Constitutive heterochromatin was visualized using C-banding following Sumner [84]. 

### 4.5. Fluorescence In Situ Hybridization (FISH) for Repetitive DNA Mapping

The *5S* rDNA probe included the *5S* rDNA coding region, with 120 base pairs (bp), and the 200 bp long non-transcribed spacer (NTS) [85]. The *18S* rDNA probes were obtained by PCR using primers described in Cioffi et al. [86]). The *18S* and *5S* rDNA probes were labeled directly with the Nick-translation labeling kit (Jena Bioscience, Jena, Germany), where *18S* rDNA was labeled with Atto488 (green fluorescence) and *5S* rDNA with Atto550 (red fluorescence), according to the manufacturer’s manual. Since karyotypes of both Australian species contain the highest proportion of metacentric (m) chromosomes among arowana species, telomeric (TTAGGG)_n_ sequences were mapped to track the possible fusion events using the PNA Telomere FISH Kit/Cy3 (DAKO, Glostrup, Denmark). FISH was conducted under high stringency conditions as described in Yano et al. [87].

### 4.6. Comparative Genomic Hybridization (CGH)

The total genomic DNAs (gDNAs) were extracted from liver tissue using the standard phenol-chloroform-isoamyl alcohol method [72]. Two different experimental designs were used in this study: (1) In a set of interspecific comparative experiments, the gDNA of *S. leichardti* was co-hybridized subsequently with the gDNA of each arowana species under study against the background of female chromosomes of *S. leichardti*. The gDNA of *S. leichardti* was directly labeled with Atto550 using a Nick-translation labeling kit (Jena Bioscience), while the gDNAs of all other arowana species were labeled with Atto488 also using a Nick-translation labeling kit (Jena Bioscience). In all experiments, C0t-1 DNA (i.e., a fraction of genomic DNA enriched for highly and moderately repetitive sequences) was used and prepared according to Zwick et al. [88], for blocking common genomic repetitive sequences. The final hybridization mixture for each slide (20 µL) was composed of 500 ng of *S. leichardti* gDNA, 500 ng of the gDNA of compared arowana species, and 15 µg of unlabeled female-derived C0t-1 DNA from the compared species, all resuspended together in the hybridization buffer containing 50% formamide, 2 x SSC, 10% SDS, 10% dextran sulfate, and Denhardt’s reagent (pH 7.0). The chosen ratio of probe versus the C0t-1 DNA amount was based on the experiments performed in our previous studies in fishes [87,89,90,91,92,93,94]. (2) Since a presence/absence polymorphism for conspicuous secondary constriction and a corresponding constitutive heterochromatin block appeared in a single chromosomal pair harboring in both male and female individuals of *S. formosus*, we compared the genomes of individuals carrying versus not carrying such a conspicuous block. The gDNA of individuals bearing the block was labeled with Atto550, and the gDNA of individuals lacking this region was labeled with Atto488 via Nick-translation as described above. The final probe cocktail for each experiment was composed of 500 ng of each probe and 15 µg of C0t-1 DNA of the respective individuals, diluted in the same hybridization buffer as described above. The CGH experiments were performed according to Symonová et al. [95].

### 4.7. Microdissection and the Preparation of Chromosome Painting Probes

Fifteen copies of the first chromosome pair of *O. ferreirai* and 12 copies of the largest acrocentric (that harbors a conspicuous secondary constriction) present in *S. formosus* were isolated via microdissection and amplified using the procedure described in Yang et al. [96]. We referred to these probes as OFE-1 and SFO-A, and they were labeled with Spectrum Green-dUTP and Spectrum-Orange-dUTP (Vysis, Downers Grove, IL, United States), respectively, in a secondary DOP PCR using 1µL of the primarily amplified product as a template DNA, also following Yang et al. [96]. Chromosomal preparations of all Arowana species were used for the Zoo-FISH experiments with both WCP probes. The hybridization was performed following the protocol described in Yano et al. [87,93].

### 4.8. Image Analysis and Processing

At least 30 metaphase spreads per individual were analyzed to confirm the 2*n*, karyotype structure, and FISH results. Images were captured using an Olympus BX50 microscope (Olympus Corporation, Ishikawa, Japan) with CoolSNAP and the images were processed using the Image Pro Plus 4.1 software (Media Cybernetics, Silver Spring, MD, USA). Chromosomes were classified as metacentric (m), submetacentric (sm), subtelocentric (st), or acrocentric (a), according to their arm ratios [97].

## Figures and Tables

**Table 1:** Collection sites of the Arowana species analyzed, with the sample sizes (N).

Species	Sampling Site	N
*Osteoglossum bicirrhosum*	Confusão Lake, Araguaia River.	(12♀ 11♂)
*Osteoglossum bicirrhosum*	Catalão Lake, Solimões River	(12♀ 11♂)
*Osteoglossum ferreirai*	Negro River (Amazon River Basin)	(15♀ 19♂)
*Scleropages formosus* (Super Red variety)	Origin unknown, Aquarium trade	(03♀ 02♂)
*Scleropages jardinii*	Corroboree Billabong, Mary River	(05♀ 03♂)
*Scleropages leichardti*	Fitzroy River via aquarium trade	(03♀ 04♂)

## Supplementary Materials

Supplementary materials can be found at https://www.mdpi.com/1422-0067/20/17/4296/s1.
