# Supplementary figures and images for: Deciphering the Evolutionary History of Arowana Fishes (Teleostei, Osteoglossiformes, Osteoglossidae): Insight from Comparative Cytogenomics
Source: Int J Mol Sci. 2019 Sep 2;20(17):4296. doi: 10.3390/ijms20174296 (PMC6747201; doi:10.3390/ijms20174296)

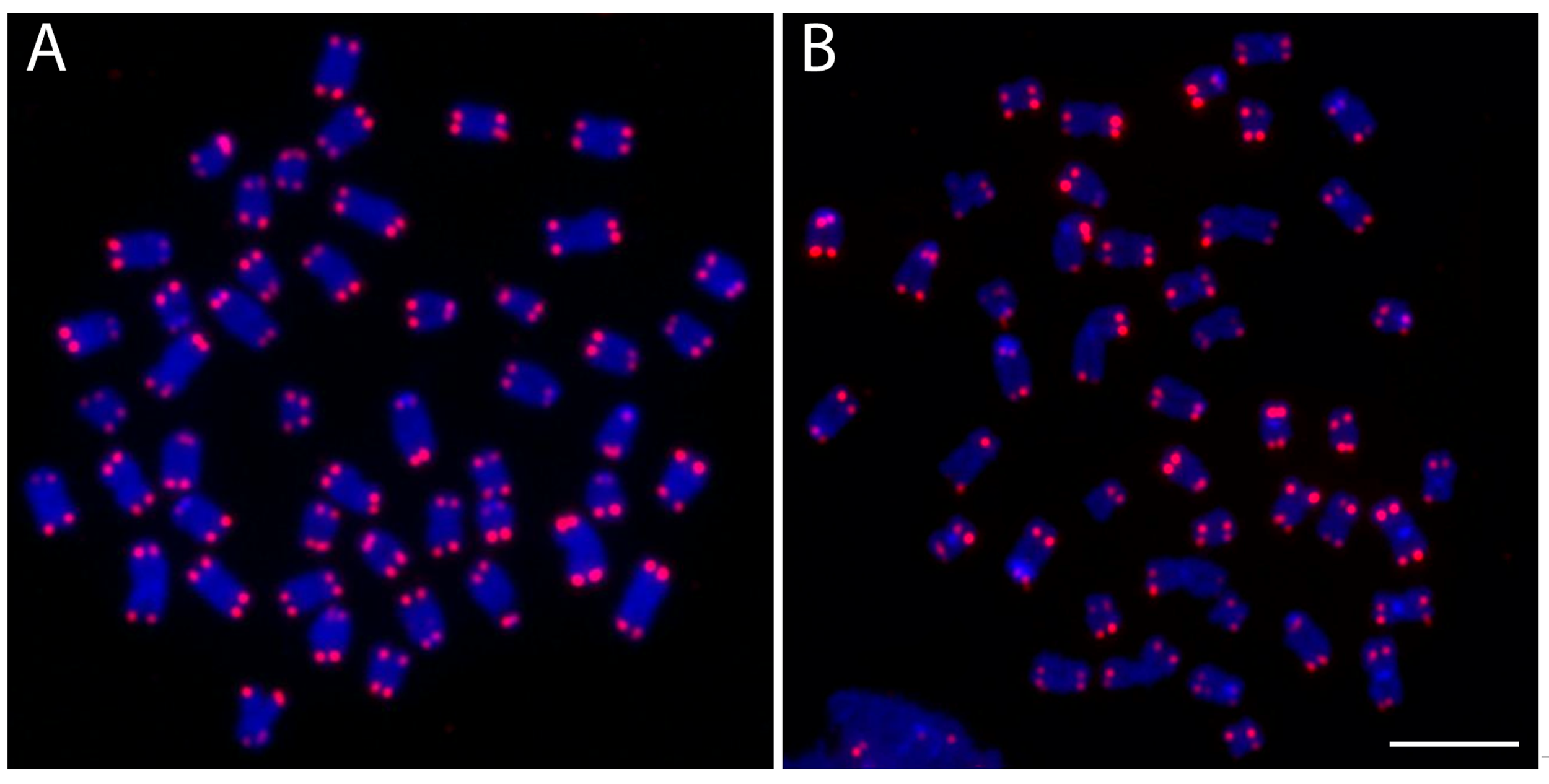

Supplement: Supplementary file 1 [file ijms-20-04296-s001.zip › ijms-583796 suppl/S1_fig.tif]

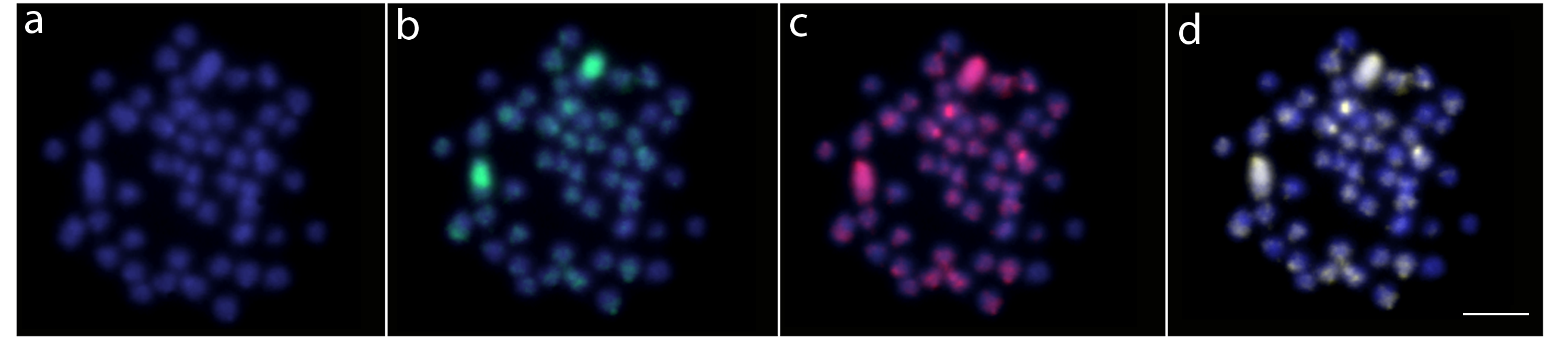

Supplement: Supplementary file 1 [file ijms-20-04296-s001.zip › ijms-583796 suppl/S2_fig.tif]
